# Codon usage and codon context bias in *Xanthophyllomyces dendrorhous*

**DOI:** 10.1186/s12864-015-1493-5

**Published:** 2015-04-13

**Authors:** Marcelo Baeza, Jennifer Alcaíno, Salvador Barahona, Dionisia Sepúlveda, Víctor Cifuentes

**Affiliations:** Departamento de Ciencias Ecológicas, Facultad de Ciencias, Universidad de Chile, Las Palmeras 3425, Casilla 653, Santiago, Chile

**Keywords:** Codon usage bias, *X. dendrorhous*, Codon context bias

## Abstract

**Background:**

Synonymous codons are used differentially in organisms from the three domains of life, a phenomenon referred to as codon usage bias. In addition, codon pair bias, particularly in the 3’ codon context, has also been described in several organisms and is associated with the accuracy and rate of translation. An improved understanding of both types of bias is important for the optimization of heterologous protein expression, particularly in biotechnologically important organisms, such as the yeast *Xanthophyllomyces dendrorhous,* a promising bioresource for the carotenoid astaxanthin. Using genomic and transcriptomic data, the codon usage and codon context biases of *X. dendrorhous* open reading frames (ORFs) were analyzed to determine their expression levels, GC% and sequence lengths. *X. dendrorhous* totiviral ORFs were also included in these analyses.

**Results:**

A total of 1,695 *X. dendrorhous* ORFs were identified through comparison with sequences in multiple databases, and the intron-exon structures of these sequences were determined. Although there were important expression variations among the ORFs under the studied conditions (different phases of growth and available carbon sources), most of these sequences were highly expressed under at least one of the analyzed conditions. Independent of the culture conditions, the highly expressed genes showed a strong bias in both codon usage and the 3’ context, with a minor association with the GC% and no relationship to the sequence length. The codon usage and codon-pair bias of the totiviral ORFs were highly variable with no similarities to the host ORFs.

**Conclusions:**

There is a direct relation between the level of gene expression and codon usage and 3′ context bias in *X. dendrorhous*, which is more evident for ORFs that are expressed at the highest levels under the studied conditions. However, there is no direct relation between the totiviral ORF biases and the host ORFs.

**Electronic supplementary material:**

The online version of this article (doi:10.1186/s12864-015-1493-5) contains supplementary material, which is available to authorized users.

## Background

With the exception of methionine and tryptophan, amino acids are encoded by two to six synonymous codons according to the standard genetic code, and degenerate codons are used at different frequencies, a phenomenon known as codon usage bias (CUB) [1]. Several biological factors, such as the gene GC composition and length, mutation frequency and pattern, gene expression level, tRNA abundance, gene translation initiation signals and protein structure, influence the CUB [2-9].

The existence of CUB has been described in metazoans [10], *D. melanogaster* [11], bacteria [12,13], insects [14], archaea [15] and viruses [16-18]. It has been proposed that viral genomes adapt to the host codon usage to efficiently use the host’s translational resources [19,20]. Previous studies have reported interspecies or even intraspecies differences between highly and poorly expressed genes, likely associated with translational efficiency [21,22]. Highly expressed genes typically exhibit higher bias in synonymous codon usage, and it has been proposed that mutation pressure and natural selection are the major forces influencing this phenomenon, favoring translationally superior codons [23-27]. Thus, the most optimal codons are significantly more represented in highly expressed genes than in poorly expressed genes [28,29]. In addition to CUB, codon context bias reflects preferences related to the sequentiality of a pair of codons (codon pair). Codon context bias is likely associated with the accuracy of decoding, indicating the ability of the translational machinery to detect codon pairs present at ribosomal decoding sites [30-33]. One hypothesis is that translation rates are influenced by the compatibilities of adjacent tRNAs at the A- and P-sites on the surface of translating ribosomes. The results of a recent *in vivo* study suggested that the codon context primarily influences the speed at which proteins are synthesized in *E. coli* [34]. Preferred and avoided codon pairs have been observed in the three domains of life, and it has been reported that 3′ codons primarily show selective effects on the codon context [35].

Both CUB and codon context bias analyses have been recommended for the optimization of heterologous gene expression, as parameters that significantly favor gene expression [36]. Thus, knowledge of the CUB and codon context bias is of critical interest for genetic improvement when heterologous expression is used to favor the productivity of biotechnologically important microorganisms. The basidiomycetous yeast *Xanthophyllomyces dendrorhous* is relevant to biotechnology, as this microorganism synthesizes the carotenoid astaxanthin. This pigment has strong antioxidant properties beneficial for human health, including potential benefits for the treatment of degenerative diseases [37]. In addition, astaxanthin is commonly used in aquaculture for the pigmentation of the flesh of salmonid fishes, which is a considerably important factor in this industry. Although *X. dendrorhous* is a promising source of natural astaxanthin, natural production in wild-type strains is not sufficient to be economically competitive against the chemical synthesis of this pigment. Therefore, considerable effort has been made to improve the production of carotenoids in *X. dendrorhous*, including culture optimization, classical random mutagenesis and metabolic engineering approaches (reviewed in [38]). Unfortunately, the molecular tools to genetically modify this yeast remain scarce [39], limiting the number of potential modifications that may be of interest. Thus, knowledge of the CUB and codon context bias for this yeast would be a pivotal contribution to the design of new metabolic engineering strategies to improve astaxanthin biosynthesis in this organism. In addition, totiviruses have recently been identified in *X. dendrorhous* strain UCD 67–385 [40]; unlike mammalian viruses, these viruses lack an extracellular infection route and are cytoplasmically transmitted.

Although the codon usage of *X. dendrorhous* has been previously described, the analysis was performed using only ten ribosomal genes [41]. However, the current application of next-generation technologies has provided additional information to conduct more representative studies. In the present study, we evaluated the codon usage and codon context bias of multiple *X. dendrorhous* genes using genomic and transcriptomic data obtained from the yeast cultured with two different carbon sources (glucose and succinate) during two different phases of growth (exponential and stationary). The level of gene expression was included as a parameter in these analyses for the comparison of codon usage and codon context biases among highly and lowly expressed genes, and the gene expression was also compared against totivirus genes resident in this yeast.

## Results

### Open reading frame (ORF) identification and expression analysis

**Figure 1:**
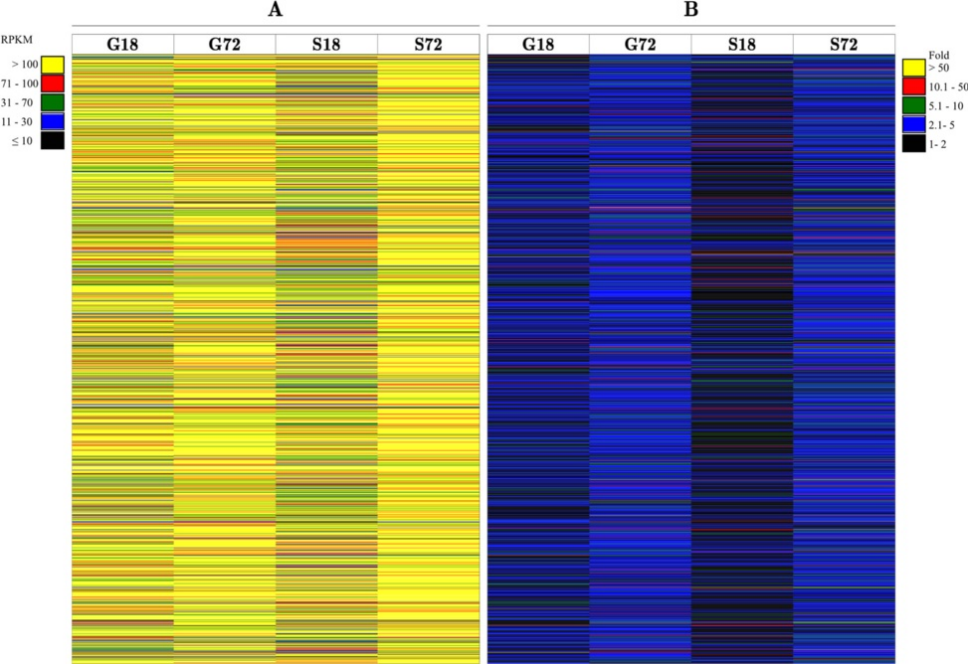
Expression levels of the 1,695 ORFs from *X. dendrorhous.* **A**: Expression level of each ORF in RPKM. The color code scale represents the RPKM values as indicated in the figure. **B**: Comparison of the expression levels of each ORF between the four analyzed conditions. The RPKM values of each ORF in each condition were normalized to the lowest RPKM value observed for each ORF. The color code scale represents the expression fold-changes as indicated in the figure. In both panels, G18 and S18 represent the early log-phase of growth in cultures with glucose or succinate as the sole carbon source, respectively; G72 and S72 correspond to the initial stationary phase of growth with glucose or succinate as the sole carbon source, respectively.

The *X. dendrorhous* strain UCD 67–385 was grown in minimal media supplemented with glucose or succinate as the sole carbon source, and the cells were collected at the early exponential (~18 h) and initial stationary (~72 h) phases of growth, generating a total of four different conditions (G18, S18, G72 and S72: *G*lucose or *S*uccinate and *18* or *72* h of culture). Total RNA was purified from the yeast pellets, and the quality of samples was assessed and sequenced using the Illumina GAII and HiSeq platforms. Open reading frames (ORFs) of at least 300 bp in length were predicted using transcriptome contigs, and subsequently these sequences were mapped to five genomic scaffolds of 1.1 to 2.4 Mbp in length (approximately 8.1 Mbp in total). Only the mapped ORFs identified under the four conditions were analyzed, and ORFs showing 100% identity with genome sequences, including a well-defined exon-intron structure, were selected and compared with the database using the Blast2GO server [42]. Among the 2,434 sequences analyzed, 1,695 sequences showed positive Blastx hits to at least one conserved protein domain in the InterPro database [43] (maximum e-value 10^−85^) (Additional file 1). The remaining 739 sequences with no Blastx hits were not included in the following analyses. In each of the four conditions, the transcriptional levels of each ORF were quantified as reads per kilobases per million mapped reads (RPKM) as previously described [44]. In general, the analyzed ORFs were highly expressed (Figure 1A), and among the four conditions, the percentages of ORFs with RPKM values considered as low- to moderate- (1–30 RPKM), quite high- to high- (31–100 RPKM) and over- (>100 RPKM) expressed, ranged from 2.9 to 10.7, 14.7 to 31 and 58.3 to 82.4%, respectively. The major percentages of over-expressed ORFs were observed after culturing *X. dendrorhous* in both carbon sources for 72 h, with 82.4% for succinate and 75.8% for glucose. Considering the highest RPKM value for each ORF observed among the four conditions, the percentages of low- to moderate-, quite high- to high- and over-expressed ORFs were 1.6, 10 and 88.4%, respectively. Variations in the expression levels of each ORF were determined by normalizing the RPKM value of each ORF in the reference condition to the lowest RPKM value of the respective ORF among the four conditions. The majority of the ORFs showed considerable variations in expression among the four conditions, although most of the genes were over-expressed (Figure 1B). Smaller differences in the RPKM values were observed after 18 h of culture, and the lowest values were observed using succinate as the sole carbon source. Taking the ratio between the highest and the lowest RPKM value of each ORF among the four conditions as a fold-change in expression, the percentages of ORFs with 1–2, 2.1-5, 5.1-10, 10.1-50 and >50-fold-changes, were 21, 59, 14, 5 and 1%, respectively. The ten ORFs showing the highest expression levels and the highest fold-changes, without considering the ribosomal genes, are listed in Table 1.

**Table 1:** ***X. dendrorhous*** **ORFs with the highest expression levels and maximum expression fold-changes among the four conditions**

**ORF**	**RPKM**	**ORF**	**Fold-change**
Cytochrome c oxidase family protein	38,981 (S18)	MFS polyamine transporter	273 [S72/G72]
Carbohydrate-binding module family 13 protein	34,225 (S72)	Glycoside hydrolase family 3 protein	236 [G18/S72]
Hsp10-like protein	26,138 (S18)	NAD-dependent formate dehydrogenase	231 [G18/G72]
Lipid droplet-associated perilipin protein	22,038 (G18)	DUF895 domain membrane protein	101 [S72/G18]
F-type H -transporting ATPase subunit J	21,800 (S72)	Carbohydrate-binding module family 13 protein	98 [G18/S72]
FK506 binding protein	20,367 (S18)	Isocitrate lyase	85 [G18/S72]
Probable GRX1-glutaredoxin	16,503 (S18)	Transcriptional regulator	83 [G72/G18]
Eukaryotic ADP ATP carrier	16,209 (G18)	Hsp10-like protein	82 [G18/S72]
Glucose oxidase	11,505 (S72)	MFS general substrate transporter	82 [G18/S72]
D-lactate dehydrogenase oxidoreductase protein	9,983 (G72)	NADPH2 dehydrogenase	72 [S72/S18]

( ): condition in which the highest RPKM value was observed. [ ]: condition in which the highest/lowest RPKM values were observed.

### Codon usage bias analysis

**Figure 2:**
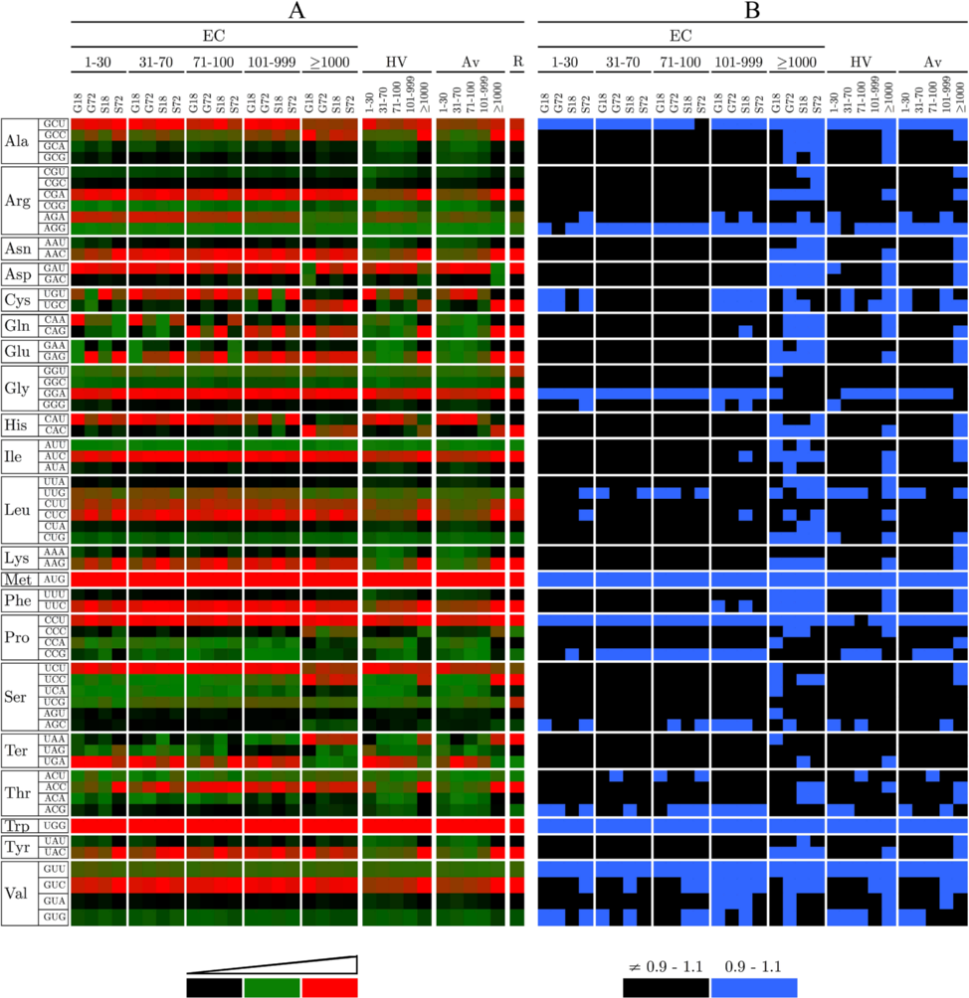
Codon usage in *X. dendrorhous* ORFs classified according to their expression level. The ORFs were classified according to the RPKM values, considering the expression levels of these genes in *E*ach *C*ondition (EC grouping), the *H*ighest RPKM *V*alue registered among the four conditions (HV grouping) and the *A*verage RPKM *V*alue for the four conditions (AV grouping). The ribosomal ORFs (R) were grouped independently. The RPKM values ranged from 1–30, 31–70, 71–100, 101–999 and ≥ 1,000. G18 and S18 represent 18 h of culture using glucose or succinate as the sole carbon source, whereas G72 and S72 correspond to 72 h of culture using glucose or succinate as the sole carbon source, respectively. **A**: Codon usage for each amino acid. The continuous color scale indicates the most common, intermediate and less frequent codons in red, green and black, respectively. **B**: Codon usage comparison between ORFs under different classification criteria against the ribosomal codon usage. The figure illustrates the RSCU ratios of each codon among the different classification criteria and the ones from the ribosomal ORFs. Ratios between 0.9 and 1.1, indicating similar RSCU values, are represented in blue, whereas ratios outside this range are represented in black.

To analyze the *X. dendrorhous* CUB, the ORFs were classified according to their expression level under *E*ach *C*ondition (EC grouping), with RPKM values ranging from i) 1–30, ii) 31–70, iii) 71–100, iv) 101–999 and v) ≥1,000. The ribosomal ORFs commonly used as references for highly expressed genes were grouped separately (R grouping). However, as an ORF can be poorly expressed under one condition but highly expressed under another, the ORFs were also classified using the same RPKM value ranges but only considering the *H*ighest RPKM *V*alue observed among the four conditions (HV grouping). In addition, the ORFs were also classified according to the *A*verage RPKM *V*alue among the four conditions (AV grouping). The analysis of relative synonymous codon usage was performed for each group within a classification using the CodonW program/server/software (http://mobyle.pasteur.fr/cgi-bin/portal.py#forms::CodonW, [24]), and the results are illustrated in Figure 2. Although a direct relation between the expression level and the codon usage was observed in the EC grouping, some variations were observed, depending on the condition (Figure 2A). However, a clearer tendency was observed in the HV and AV grouping, where ORFs with higher expression levels showed a greater preference for some codons including the ribosomal ORFs. Using the codon bias of ribosomal ORFs as a reference, a pattern similar to that of the highly expressed genes in the three different groupings was observed, but this tendency was clearly detected when the HV and AV grouping was compared (Figure 2B). This finding might reflect the differential expression of the analyzed ORFs under different conditions, affecting the number of ORFs in each group (Table 2). The relative synonymous codon usage (RSCU) for the ribosomal ORFs, the highly expressed genes defined in the HV grouping and the ribosomal ORFs are shown in Table 3.

**Table 2:** **ORFs classified according to each condition (G18, G72, S18 and S72), the highest registered (HV) and average (AV) RPKM values**

	RPKM (N; X)*					
	**EC**				**HV**	**AV**
**Group**	**G18**	**G72**	**S18**	**S72**		
1-30	140; 17	72; 20	185; 17	49; 16	27; 20	65; 19
31-70	256; 51	183; 51	310; 50	118; 53	75; 53	168; 54
71-100	174; 85	157; 84	214; 84	133; 86	94; 86	158; 86
101-999	983; 309	1,086; 336	839; 315	1,257; 314	1,202; 350	1,148; 317
≥1,000	142; 5,879	197; 3,063	147; 7210	138; 3516	297; 5,407	156; 4,600

*N: number of ORFs; X: RPKM average value from the ORFs in each group.

**Table 3:** **Relative synonymous codon usage of ribosomal and highly expressed ORFs of the** ***X. dendrorhous***

**AA**	**Cod**	**RSCU**	**AA**	**Cod**	**RSCU**	**AA**	**Cod**	**RSCU**	**AA**	**Cod**	**RSCU**
Ala	GCU	1.22 (1.40)	Gln	CAA	0.83 (0.90)	Leu	CUC	1.83 (1.53)	Ser	AGU	0.40 (0.61)
Ala	GCC	1.91 (1.22)	Gln	CAG	1.17 (1.10)	Leu	CUA	0.22 (0.49)	Ser	AGC	0.65 (0.63)
Ala	GCA	0.50 (0.74)	Glu	GAA	0.56 (0.84)	Leu	CUG	0.95 (0.88)	TER	UAA	1.38 (1.03)
Ala	GCG	0.37 (0.64)	Glu	GAG	1.44 (1.16)	Lys	AAA	0.42 (0.75)	TER	UAG	0.50 (0.81)
Arg	CGU	0.30 (0.51)	Gly	GGU	1.39 (1.12)	Lys	AAG	1.58 (1.25)	TER	UGA	1.12 (1.16)
Arg	CGC	0.25 (0.29)	Gly	GGC	0.70 (0.77)	Met	AUG	1.00 (1.00)	Thr	ACU	0.72 (1.03)
Arg	CGA	2.83 (2.10)	Gly	GGA	1.46 (1.60)	Phe	UUU	0.55 (0.74)	Thr	ACC	2.00 (1.28)
Arg	CGG	0.30 (0.72)	Gly	GGG	0.46 (0.50)	Phe	UUC	1.45 (1.26)	Thr	ACA	0.50 (0.86)
Arg	AGA	1.28 (1.48)	His	CAU	0.79 (1.06)	Pro	CCU	1.34 (1.49)	Thr	ACG	0.78 (0.82)
Arg	AGG	1.04 (0.90)	His	CAC	1.21 (0.94)	Pro	CCC	1.09 (0.77)	Trp	UGG	1.00 (1.00)
Asn	AAU	0.41 (0.68)	Ile	AUU	0.60 (0.85)	Pro	CCA	0.56 (0.87)	Tyr	UAU	0.41 (0.80)
Asn	AAC	1.59 (1.32)	Ile	AUC	2.16 (1.82)	Pro	CCG	1.01 (0.87)	Tyr	UAC	1.59 (1.20)
Asp	GAU	0.99 (1.13)	Ile	AUA	0.24 (0.32)	Ser	UCU	1.08 (1.46)	Val	GUU	1.04 (1.10)
Asp	GAC	1.01 (0.87)	Leu	UUA	0.19 (0.42)	Ser	UCC	1.77 (1.18)	Val	GUC	1.76 (1.59)
Cys	UGU	1.04 (1.03)	Leu	UUG	0.91 (1.28)	Ser	UCA	0.45 (0.92)	Val	GUA	0.53 (0.48)
Cys	UGC	0.96 (0.97)	Leu	CUU	1.90 (1.40)	Ser	UCG	1.65 (1.19)	Val	GUG	0.67 (0.82)

The RSCU values, including the ribosomal ORFs and the ORFs in the ≥1,000 group according to the HV grouping, are shown in parenthesis.

In addition, the ORFs were classified according to sequence length and GC% to analyze the CUB. When the ORFs were grouped according to sequence length, all groups showed similar codon usage, and only the shorter sequences, ranging from 300 to 499 bp, showed some differences with the larger ORFs (Additional file 2 A and B). A direct relation between the CUB and the GC% was observed, with a greater bias in ORFs with a higher GC content (Additional file 2 C). Greater differences in the RSCU ratios between the data for each GC% group and that for the group with 54% GC were observed, whereas the differences in GC% in the ORFs increased (Additional file 2 D).

**Figure 3:**
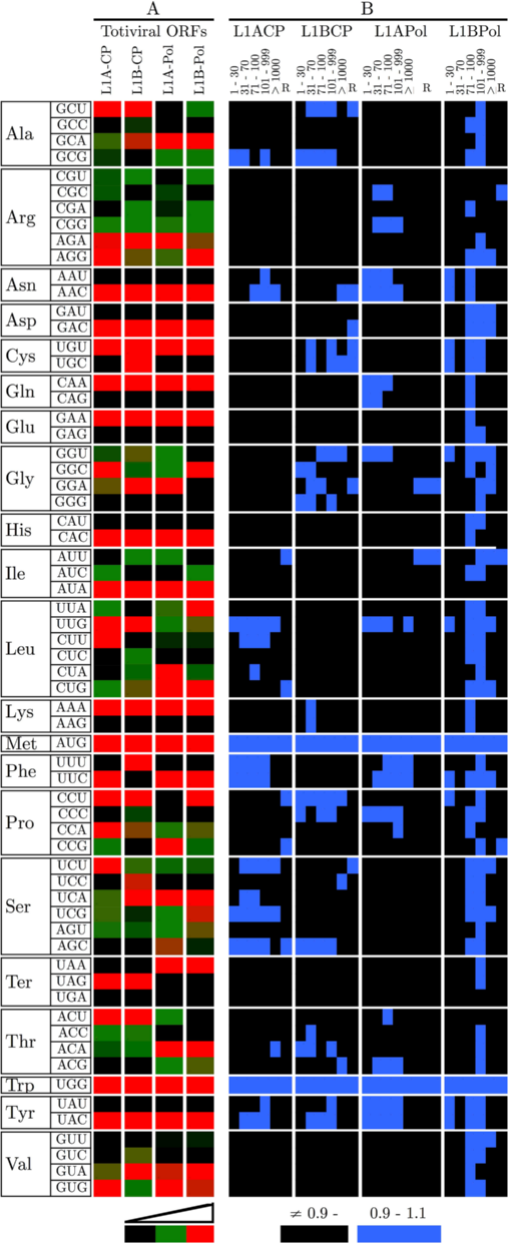
Codon usage of the *X. dendrorhous* totivirus genomes. L1A-CP and L1B-CP correspond to the ORFs of the capsid protein, and L1A-Pol and L1B-Pol are the ORFs of the polymerases from XdV-L1A and XdV-L1B, respectively. **A**: Codon usage of the totiviral ORFs. The continuous color scale indicates the most common, intermediate and less frequent codons in red, green and black, respectively. **B**: Graphical representation of the RSCU ratios from each totiviral ORF and from each expression level group in the HV- and ribosomal- (R) groupings. Ratios between 0.9 and 1.1 are represented in blue, indicating similarity, whereas ratios outside this range are represented in black.

We also specifically analyzed the codon usage of the *X. dendrorhous* viral ORFs from totiviruses XdV-L1A and XdV-L1B [40]. As shown in Figure 3A, the codon usage for a majority of the amino acids was quite different among the totiviral ORFs. Compared with the host, only the XdV-L1B totiviral polymerase ORF was similar to the highly expressed *X. dendrorhous* ORFs, whereas the remaining totiviral ORFs did not show similarities with the lowly or highly expressed ORFs from *X. dendrorhous* (Figure 3B).

### Codon context bias analysis

**Figure 4:**
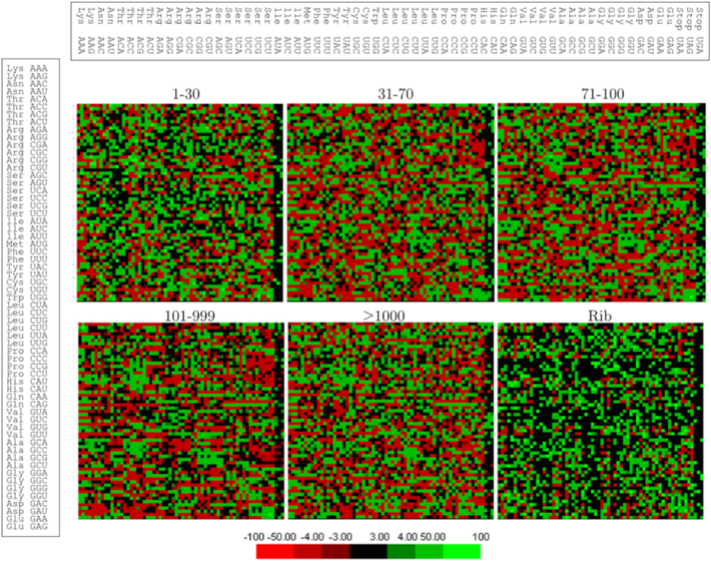
3′ codon context in *X. dendrorhous* ORFs. The ORFs were classified in six groups (1–30, 31–70, 71–100, 101–999, ≥1000 and R) according to the HV grouping, including the ribosomal group (R). The upper bar indicates the amino acid codons in the 3′ position, whereas the left bar indicates the reference amino acid codons for the six groups. Good context (the 3′ codons appear more frequently than expected) is indicated as positive values, and bad context (3′ codons appear less frequently than expected) is indicated as negative values. Values between −3 to +3 are not statistically significant (no bias).

**Figure 5:**
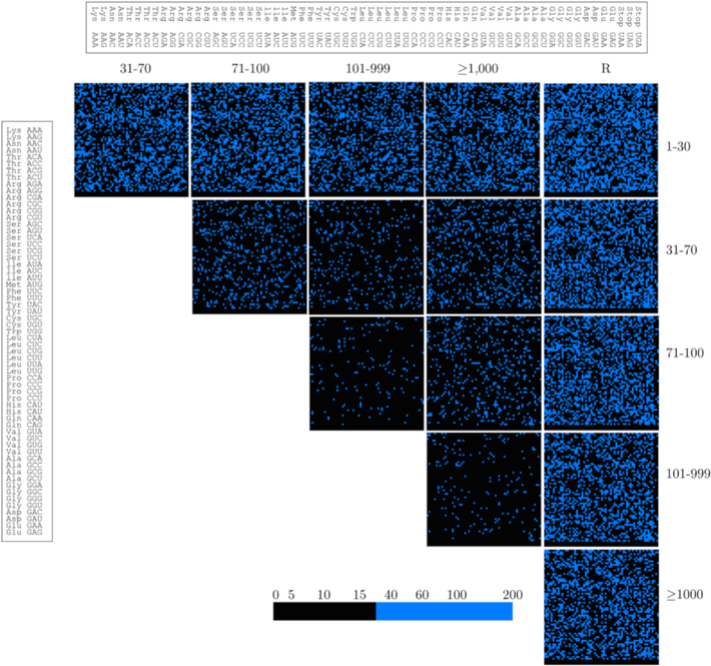
Differential display maps of codon context comparison among ORFs with different expression levels. Comparison of the 3′ codon context between groups of ORFs with different expression levels (RPKM: 1–30, 31–70, 71–100, 101–999 and ≥1,000) according to the HV grouping and the Ribosomal (R) ORFs. The upper bar indicates the amino acid codons in the 3′ position, and the left bar indicates the reference amino acid codons for each analysis. The color coding scale is indicated in the figure, where codons with similar residual values are indicated in black and differences are indicated in blue.

The 3′ codon context analysis was performed using the Anaconda software [45] and the HV grouping of ORFs. A 3′ codon context bias was observed in all groups, differing according to the expression level (Figure 4). When the 3′ codon context was compared among the groups, a direct relationship between the expression level and differences in the codon context was observed: ORFs with greater differences in expression level showed greater differences in the 3′ codon context, and ORFs with RPKM values of 101–999 and ≥1,000 showed more similarities (Figure 5). The top five preferred and non-preferred codon pairs in each HV group are listed in Table 4. The non-preferred codon pairs CTT-AAG and CCT-AAG appeared in five and four groups, respectively, whereas the preferred codon pairs, TCA-TCC, AAG-AAG and GAA-GAA, appeared in three groups.

**Table 4:** **Codon pairs with minimum and maximum codon context bias values**

**Groups of ORFs with different expression level**					
R**	1-30**	31-70**	71-100**	101-990**	≥1,000**
Avoided codon pairs					
*TCG-CGA; −46*	**CTT-AAG; −34**	**CCT-AAG; −34**	**CCT-AAG; −35**	**TCT-AAG; −37**	GCT-AAC; −37
*GAT-AAG; −53*	*GAG-TCC; −34*	**TCT-AAG; −33**	**TCT-AAG; −31**	*GAT-AAG; −32*	*GCT-AAG; -37*
GAG-CGA; −44	ACT-AAG; −33	**CTT-AAG; −33**	CCT-GAG; −31	**CTT-AAG; −32**	**CTT-AAG; -36**
AAC-CGA; −43	GCT-AAA; −33	*GCT-AAG; −31*	*GAG-TCC; −31*	**CCT-AAG; −32**	GGA-CTC; -35
**CCT-AAG; −45**	TCT-GAG; −32	GAG-TCT; −31	**CTT-AAG; −30**	GGT-AAG; −31	*TCG-CGA; -34*
Preferred codon pairs					
*CCG-ATA; 211*	CGT-CAA; 74	**TCA-TCC; 57**	*AAG-AAG; 61*	**GAA-GAA; 59**	*CCG-ATA; 58*
CGT-CGT; 175	GTT-CCC; 59	*AAG-AAG; 50*	**TCA-TCC; 50**	*AAG-AAG; 56*	AGA-AGA; 57
AAT-TTA; 159	GTG-TTC; 56	**GAA-GAA; 49**	**GAA-GAA; 49**	**TCA-TCC; 45**	TGT-GGA; 53
TTA-ACA; 153	AGG-AAA; 56	TCT-TCT; 45	TCG-ACC; 47	CTT-CGA; 43	CGC-TTA; 52
ATA-TCA; 145	ACT-CCT; 55	GAA-GAG; 45	GCG-CTC; 47	TCT-TCC; 41	GTA-AGC; 50

Codon pairs shared by two groups are in italics, and those shared by three or more groups are in bold. The residual value is given after the semicolon; positive values indicate good context, and negative values indicate bad context. *Ribosomal ORFs; **: RPKM value ranges according to HV grouping.

A codon context bias in all groups classified according to the ORFs GC% was observed (Additional file 3 A). The comparison analysis revealed that groups having nearly 50% GC content showed a similar 3′ codon bias. For example, groups having 53 and 54% GC content were more different than groups with 49 and 50% or 50 and 51% GC content (Additional file 3 B). In the case of the ORF length, a 3′ codon context bias was observed in all groups (Additional file 4 A), and these findings were similar among groups of ORFs with 500 or more bases. Shorter ORFs of 300 to 499 bases showed a different 3′ codon context bias (Additional file 4 B).

The analysis of the 3′ codon context of the totiviral ORFs showed differences between the capsid protein ORFs of XdV-L1A and XdV-L1B and the viral polymerase ORFs from both totiviruses (Additional file 5). When the totiviral ORFs were compared with the cellular ORFs, no similarities were observed between poorly or highly expressed ORFs (Additional file 5).

## Discussion

In the present study, based on genomic and transcriptomic data, 1,695 ORFs were selected from the *X. dendrorhous* strain UCD 67–385, represented in two phases of growth for the yeast cultured using two different carbon sources, glucose or succinate. Furthermore, these ORFs encode a polypeptide with conserved domains listed in the InterPro database and showed a well-defined exon-intron structure. The expression analysis indicated that a majority of the analyzed genes were highly expressed under the four conditions, particularly in older cultures (glucose and succinate, 72 h). Among the four conditions, lower expression levels were observed for the 18-h cultures containing succinate as the sole carbon source, and the expression of a majority of the genes increased from 18 to 72 h of culture in both carbon sources. As several factors influence the CUB, in the present study, we examined the *X. dendrorhous* CUB according to the gene expression level, sequence length and GC% of the ORF. Clearly defined ORFs were classified according to these parameters, and the 3′ codon context was also analyzed.

In the first analyses, the ORFs were classified into groups according to the expression levels observed under each of the four conditions, independently exhibiting CUB differences among the groups without a clear relationship between the CUB and the expression level. The highest or average expression levels among the four conditions were used to group the ORFs according to their expression level. In these cases, a direct relationship between the CUB and the expression level was observed, and the highly expressed genes showed a major bias, with the exception of the Asp, Cys and His codons. A comparison of the CUB among all groups, based on the expression levels, revealed that the codon usage was similar among genes with similar levels of expression. Although this finding seems rather obvious, the gene expression varied under different conditions; therefore, to classify a gene as lowly or highly expressed based on only one culture condition and state of growth could lead to errors in gene classification and analysis.

Previously, the CUB for the *X. dendrorhous* strain CBS 6938 was described using ten ribosomal genes [41]. However, when considering a higher number of highly expressed genes from another strain (UCD 67–385), important differences were detected. In the previous study, the usage of the codons GCG (Ala), CGC (Arg), GGG (Gly), AUA (Ile), CUA and CUG (Leu), UUU (Phe), UGA (TER) and UAU (Tyr) in *X. dendrorhous* was not observed; however, in the present study, we observed that although these codons are not the preferred codons for each amino acid or for a stop codon, these codons are indeed used in *X. dendrorhous*. In addition, there are more than ten-fold differences in the RSCU values determined for codons GCA (Ala), AGA (Arg), CAA (Gln), GAA (Glu), CAU (His), AGC (Ser) and GUA (Val) between the previous and present studies.

The results obtained from transcriptomic analyses are consistent with the results of previous expression studies. For example, the expression of the genes encoding astaxanthin synthase and phytoene-beta carotene synthase was quantified using RT-qPCR in *X. dendrorhous* cultured in glucose and succinate as the sole carbon sources [46], and similar results were observed. The direct relation between the gene expression levels and codon usage biases observed in *X. dendrorhous* was also consistent with that of other organisms in which highly expressed genes generally show a higher synonymous codon usage bias attributed to selection for efficient translation [24,25,27,47]. Other factors, including gene length [25] and GC% [48,49], might also influence codon bias. Therefore, we analyzed the codon bias according to these parameters, but no relation was observed for gene length, and although a direct relation regarding the GC% was observed, this association was not as evident as for the gene expression level.

Information regarding the CUB is important in the field of heterologous gene expression to achieve the efficient production of recombinant proteins, for example, enzymes relevant in the biotechnology industry [50]. In recent years, it has been suggested that the codon context or codon pairs might influence translational accuracy and speed, as preferences for specific codon pairs are observed in the three domains of life, referred to as codon context bias [34,35]. Actually, the codon context bias, particularly the 3′ codon context, has been proposed to have as much or even more influence on heterologous gene expression than the CUB [36,51,52]. We observed variations in the 3′ codon context among the groups of genes with different expression levels, and we detected major differences between genes with different expression levels. When we analyzed the genes based on the GC% or sequence length, a codon context bias was observed in which genes with nearly 50% CG content had similar biases. In the case of the gene length, genes of 500 or more bp showed a similar codon context bias. In the three domains of life, the codon pairs with nnUAnn, nnGGnn, nnGnnC, nnCGCn, GUCCnn, CUCCnn, nnCnnA or UUCGnn patterns are most frequently avoided, and codon pairs with nnGCnn, nnCAnn or nnUnCn patterns are most frequently preferred [35]. In the present study, the most avoided codon pairs in *X. dendrorhous* were consistent with the described patterns, i.e., CC*UA*AG, GA*G*UC*C* and AA*C*CG*A*, and the most preferred codon pairs were GC*GC*TC, GU*U*C*C*C, AC*U*C*C*U, UC*U*U*C*U and UC*U*U*C*C (the most conserved nucleotides in each pattern are in italics).

In general, viruses do not encode tRNAs, and the synthesis of viral proteins is dependent on the host translational machinery. Thus, several virus sequences have adapted to the host codon usage, including viruses that infect humans and other mammals, particularly for highly expressed genes [19,53]. Two totivirus genomes are present in *X. dendrorhous* strain UCD 67–385 [40]; thus, we analyzed the codon usage and the 3′ codon context bias of four totiviral genes with observed variations in both types of bias in all the analyzed genes. Compared with the cellular genes, no similarities with any group classified according to expression level were observed.

## Conclusions

In general, the identified *X. dendrorhous* ORFs are highly expressed, particularly during the stationary phase of growth using succinate or glucose as the sole carbon source, and the majority of the ORFs showed considerable variations in expression under the conditions studied. The codon usage bias and the 3′ codon context bias showed a clear direct relation with the expression levels and GC% of the ORFs, but not the sequence length. However, no similarities among the totiviral and host ORFs were observed for either codon usage or 3′ codon context biases.

## Methods

### *X. dendrorhous* cultivation conditions and nucleic acid purification

The wild-type *X. dendrorhous* strain UCD 67–385 (ATCC 24230) was used for next-generation whole genome and transcriptome sequencing and analysis. The strain was cultured at 22°C with constant agitation in YM medium (1% glucose, 0.3% yeast extract, 0.3% malt extract and 0.5% peptone) for DNA extraction or in Vogel minimal medium (MM_v_) supplemented with 2% glucose or 2% succinate for RNA extraction.

The yeast RNA was purified from the early exponential (18 h) and initial stationary (72 h) phases of growth from cultures grown in MMv medium supplemented with 2% glucose or 2% succinate. After 18 h of culture in MMv medium supplemented with 2% glucose, 1% glucose remained in the medium (confirmed using the DNS method [54]).

### Purification of genomic DNA

*X. dendrorhous* DNA was isolated from protoplasts as previously described [55], resulting in a high yield of chromosomal DNA fragments larger than 50 kb. The DNA was purified using phenolic extraction (pH 8.0), including three washes with saturated phenol, three washes with phenol: chloroform: isoamyl alcohol (25: 24: 1) and one wash with chloroform: isoamyl alcohol (24: 1). Subsequently, the DNA was precipitated with 98% ethanol and washed with 70% ethanol. The dried DNA was suspended in Tris: EDTA (10: 1; pH 8.0) with 40 μg/ml of RNase A and incubated for 30 min at 37°C. The DNA was diluted five times with sterile water, and the described phenol extraction protocol was repeated. DNA samples at a 260/280 ratio of 1.7 to 1.9 and a 260/230 ratio >2, measured using a V-630 UV–vis Spectrophotometer (JASCO), were used for next-generation sequencing.

### Purification of total RNA

Total RNA was extracted from the cell pellets via mechanical rupture with 0.5 mm glass beads (BioSpec) by vortexing for 10 min, followed by the addition of Tri-Reagent (Ambion). The lysate was incubated for 10 min at room temperature, and subsequently 200 μl of chloroform per ml of Tri-Reagent was added, mixed, and centrifuged for 5 min at 4,000 x g. The aqueous phase was recovered, and two consecutive extractions with acidic phenol: chloroform (1: 1) were performed. The RNA was precipitated with two volumes of isopropanol for 10 min at room temperature, and the RNA was washed with 75% ethanol and suspended in RNase-free water. RNA samples at a 260/280 ratio >1.9, measured using a V-630 UV–vis Spectrophotometer, were used for next-generation sequencing.

### Next-generation Sequencing (NGS)

The genome of *X. dendrorhous* strain UCD 67– 385 was sequenced using the Illumina GAII Sequencing System at Amplicon Express Inc. (http://ampliconexpress.com/, Pullman, Washington, USA) and the Illumina HiSeq2000 System at Macrogen Inc. (http://dna.macrogen.com/eng/index.jsp, Seoul, Republic of Korea). Read assembly and genome and transcriptome analyses were performed using the CLC Genomics Workbench 5. We estimated that the current collection of genomic scaffolds and contigs should cover approximately 95% of the haploid genome of the yeast. For Illumina GAII genome sequencing, a 250-350-bp paired-end library and a 2,500-3,500-bp mate pair library were constructed and sequenced. In addition, 48 primer pairs across 48 gaps were designed for bulk gap closure by sequencing the PCR products with 96 primers. For Illumina HiSeq2000 genome sequencing, a 100-bp paired-end library was constructed and sequenced. The RNA samples from the 72-h culture were sequenced using a Illumina GAII, including a 250-350-bp paired-end library, and the RNA samples from the 18-h culture were sequenced using Illumina HiSeq2000, including a 100-bp paired-end library.

### ORFs and gene prediction, annotation and expression level analysis

Using the transcriptome data obtained under each condition, the open reading frames (ORFs) of at least 300 bp in length were predicted using the standard genetic code and the software Geneious® 8.0.2. ORFs that were present in yeast cultured under the four conditions were selected and mapped to five genomic scaffolds of 1,116,253; 1,334,503; 1,461,881; 1,770,274; and 2,396,803 bp. The mapped ORF sequences showing 100% identity with genome sequences, including a correct exon-intron structure, were selected, compared with the database and annotated using the Blast2GO [42] server: i) the sequences were compared against the National Center for Bioinformatics (NCBI) using the Blastx tool, with an E-value cut off of 10^−3^; ii) the blast hits of each sequence were mapped using the Gene Ontology Consortium (functional information of known gene products); iii) the GO functional annotation was completed using a cutoff value of 10^−6^; and iv) functional annotation was performed using InterPro annotations. The expression level of each ORF was calculated under each condition as reads per kilobase per million mapped reads (RPKM), as previously described [44]. The results in which the percentage of coverage of each sequence was at least 90% were used.

### Availability of supporting data

The *X. dendrorhous* ORF names, sequences, RPKM values and the viral ORF sequences used in this work are included in the Additional file 1. The Genbank accession numbers of the XdV-L1A and XdV-L1B viral genomes are [NC_020903 and JN997473], respectively.

## Additional files

Additional file 1:
**Sequences and RPKM values of ORFs used in this work.**
Additional file 2:
**Codon usage in** ***X. dendrorhous*** **ORFs grouped according to their length and GC%.** ORFs were grouped according to sequence length (A and B) and GC% (C and D); parameter ranges are indicated at the top of the figure. A and C: Codon usage for each amino acid in which the most common, intermediary and less preferred codons are represented in red, green and black, respectively, in the continuous color scale. B and D: Graphical representation of the RSCU ratios between data from each parameter ranging from 2,000 – 9,000 bp ORFs (B) or 54% GC (D). Ratios between 0.9 and 1.1 are represented in blue and indicate similarity, whereas ratios out of this range are represented in black. Additional file 3:
**3′ codon context in** ***X. dendrorhous*** **ORFs classified according to their GC content.** ORFs were classified into nine groups (44–46, 47, 48, 49, 50, 51, 52, 53 and 54) according to their GC%. The upper bar indicates the amino acid codons in the 3′ position, and the left bar indicates the reference amino acid codons for each analysis. A: 3′ codon context in ORFs classified according to their GC%. The color coding scale is indicated at the bottom of the figure, where red represents the avoided codons (negative values: bad context), and green indicates the preferred codons (positive values: good context). B: Relationship between the 3′ codon context among *X. dendrorhous* ORFs according to the GC%. The color coding scale is indicated in the figure, where black denotes similarities, and blue indicates differences. Additional file 4:
**3′ codon context in** ***X. dendrorhous*** **ORFs classified according to their length.** ORFs were classified into five groups (300–499, 500–999, 1,000-1,499, 1,500-1,999 and 2,000-9,000) according to their length in bases. A: 3′ codon context in ORFs classified according to length. The upper bar indicates the amino acid codons in the 3′ position, and the left bar indicates the reference amino acid codons. The color coding scale is indicated at the bottom of the figure, where red represent the avoided codons (negative values: bad context), and green indicates the preferred codons (positive values: good context). B: Relationship between the 3′ codon context among *X. dendrorhous* ORFs according to length. The color coding scale is indicated in the figure, where black denotes similarities, and blue indicates differences. Additional file 5:
**Relationships of the 3′ codon context of** ***X. dendrorhous*** **totiviruses.** The ORFs L1A-CP and L1B-CP encode the capsid protein, and L1A-Pol and L1B-Pol encode the polymerases from the XdV-L1A and XdV-L1B totiviruses, respectively. The upper bar indicates the amino acid codons in the 3′ position, and the left bar indicates the reference amino acid codons for each analysis. Upper panels: relationship between the 3′ codon context among four the totiviral ORFs. Middle and lower panels: relationship between the 3′ codon context with the totiviral ORFs and the ORFs with the lowest (1–30) and highest (≥1,000) expression levels in *X. dendrorhous*. The color coding scale is indicated in the figure, where black denotes similarity, and blue indicates differences.
