# Supplementary figures and images for: Codon usage and codon context bias in Xanthophyllomyces dendrorhous
Source: BMC Genomics. 2015 Apr 13;16(1):293. doi: 10.1186/s12864-015-1493-5 (PMC4404019; doi:10.1186/s12864-015-1493-5)

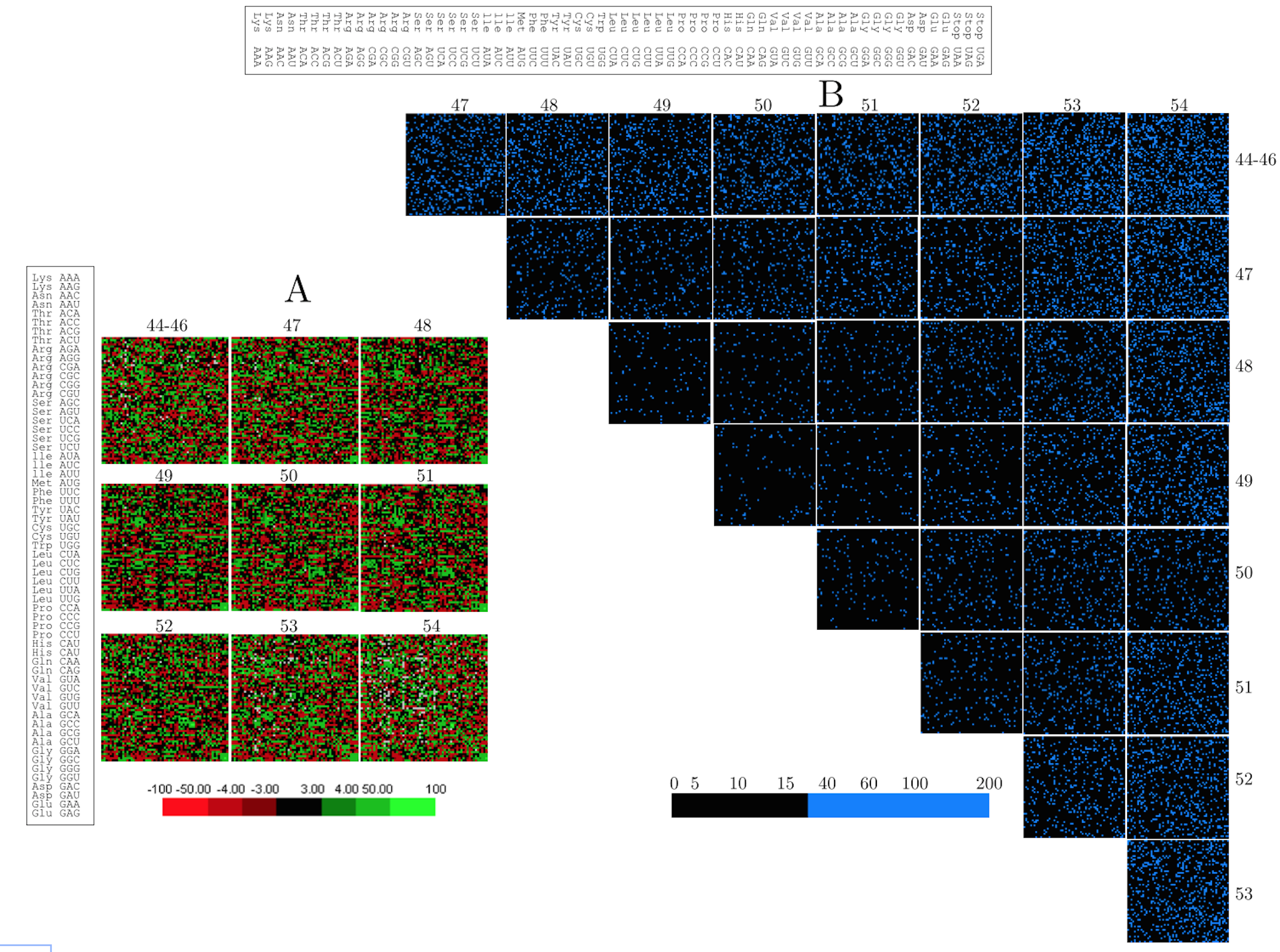

Supplement: Additional file 3: — 3′ codon context in X. dendrorhous ORFs classified according to their GC content. ORFs were classified into nine groups (44–46, 47, 48, 49, 50, 51, 52, 53 and 54) according to their GC%. The upper bar indicates the amino acid codons in the 3′ position, and the left bar indicates the reference amino acid codons for each analysis. A: 3′ codon context in ORFs classified according to their GC%. The color coding scale is indicated at the bottom of the figure, where red represents the avoided codons (negative values: bad context), and green indicates the preferred codons (positive values: good context). B: Relationship between the 3′ codon context among X. dendrorhous ORFs according to the GC%. The color coding scale is indicated in the figure, where black denotes similarities, and blue indicates differences. [file 12864_2015_1493_MOESM3_ESM.png]

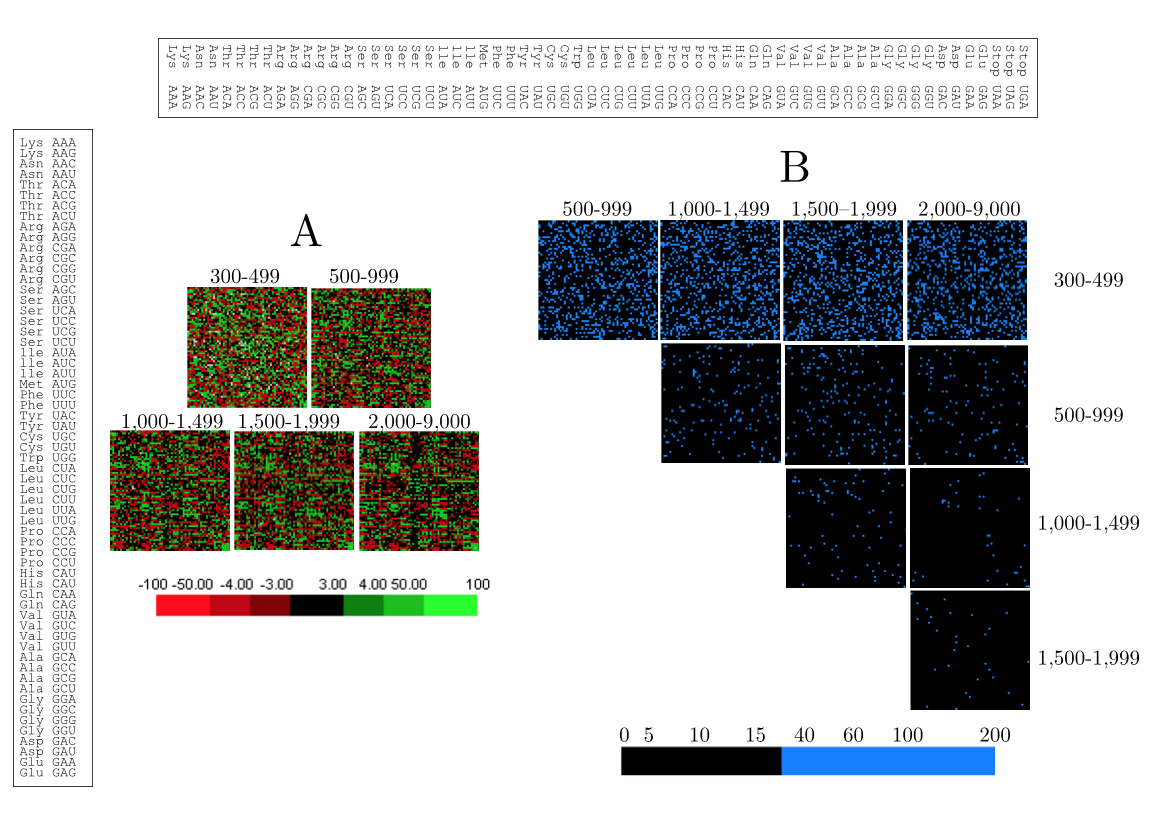

Supplement: Additional file 4: — 3′ codon context in X. dendrorhous ORFs classified according to their length. ORFs were classified into five groups (300–499, 500–999, 1,000-1,499, 1,500-1,999 and 2,000-9,000) according to their length in bases. A: 3′ codon context in ORFs classified according to length. The upper bar indicates the amino acid codons in the 3′ position, and the left bar indicates the reference amino acid codons. The color coding scale is indicated at the bottom of the figure, where red represent the avoided codons (negative values: bad context), and green indicates the preferred codons (positive values: good context). B: Relationship between the 3′ codon context among X. dendrorhous ORFs according to length. The color coding scale is indicated in the figure, where black denotes similarities, and blue indicates differences. [file 12864_2015_1493_MOESM4_ESM.png]

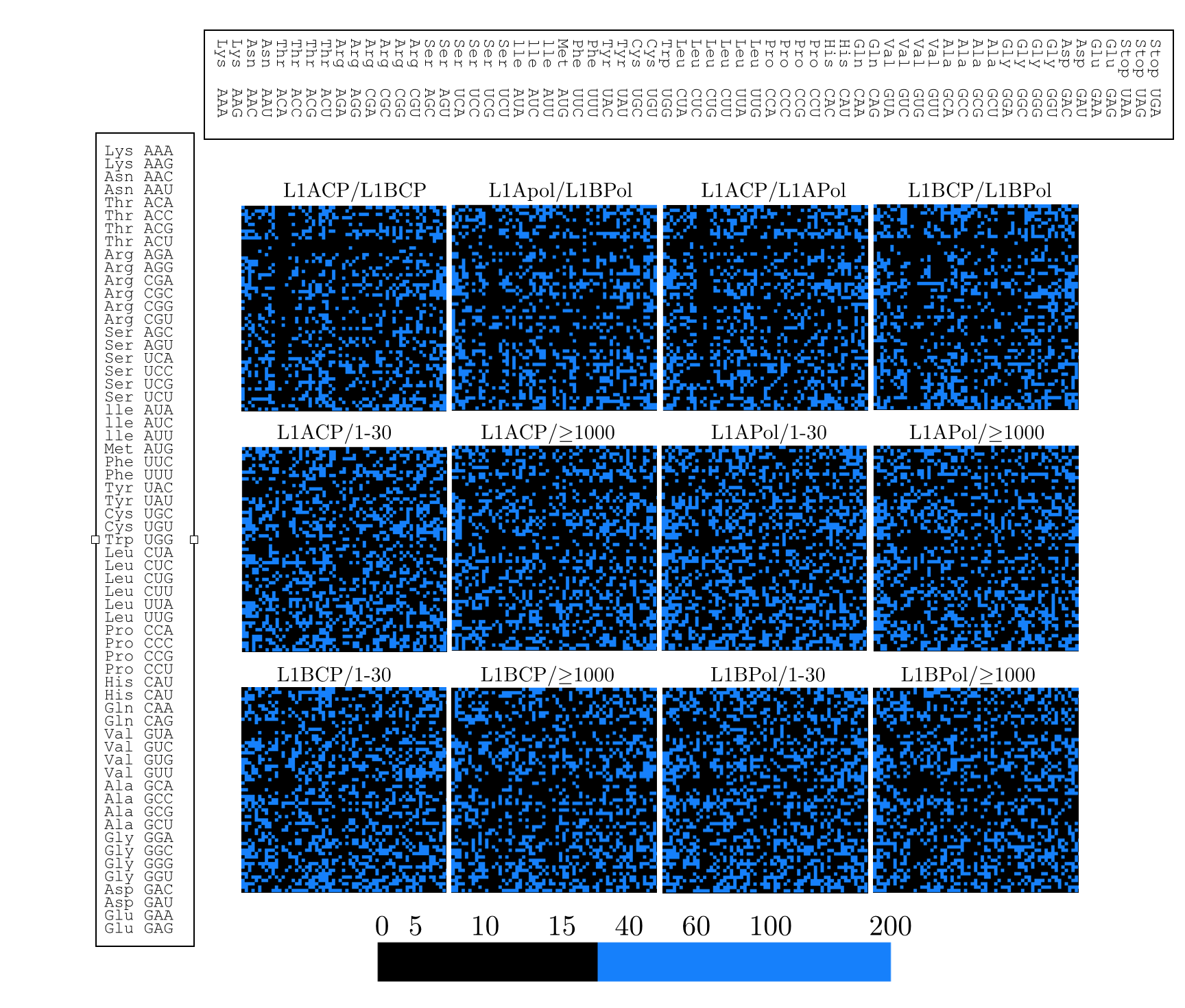

Supplement: Additional file 5: — Relationships of the 3′ codon context of X. dendrorhous totiviruses. The ORFs L1A-CP and L1B-CP encode the capsid protein, and L1A-Pol and L1B-Pol encode the polymerases from the XdV-L1A and XdV-L1B totiviruses, respectively. The upper bar indicates the amino acid codons in the 3′ position, and the left bar indicates the reference amino acid codons for each analysis. Upper panels: relationship between the 3′ codon context among four the totiviral ORFs. Middle and lower panels: relationship between the 3′ codon context with the totiviral ORFs and the ORFs with the lowest (1–30) and highest (≥1,000) expression levels in X. dendrorhous. The color coding scale is indicated in the figure, where black denotes similarity, and blue indicates differences. [file 12864_2015_1493_MOESM5_ESM.png]
